# Dynamic modulation of LH secretion by continuous kisspeptin infusion in healthy men

**DOI:** 10.1007/s42000-026-00795-y

**Published:** 2026-06-02

**Authors:** Amna Naveed, Margaret F. Lippincott, Maria Stamou, Ravi Balasubramanian, Nora K. Bryant, Stephanie B. Seminara

**Affiliations:** 1 Reproductive Endocrine Unit, Department of Medicine, Massachusetts General Hospital, 55 Fruit Street, Boston, MA 02114, USA

**Keywords:** Kisspeptin, Luteinizing hormone, Desensitization, Continuous infusion

## Abstract

**Purpose:**

Kisspeptin stimulates gonadotropin-releasing hormone (GnRH) release and, by extension, gonadotropin secretion. Although continuous kisspeptin administration has been shown to dampen kisspeptin-induced GnRH release in non-human primates [1, 2], this has not been replicated in the limited human studies reported to date. The objective of this study was to examine the effects of continuous kisspeptin administration on luteinizing hormone (LH) secretion in healthy men.

**Methods:**

Three healthy adult men received a continuous intravenous infusion of kisspeptin-10 at a dose of 12.5 mcg/kg/h for 24 h and underwent frequent blood sampling to characterize the effect on reproductive hormones.

**Results:**

Continuous administration of kisspeptin resulted in an immediate increase in LH concentrations, from a baseline of 2.8–3.8 mIU/mL to the highest values of 17.5–30.6 mIU/mL (5- to eightfold above baseline), reached within 12–20 h of infusion onset. LH concentrations subsequently declined by 13–47% from max to end of the infusion, settling at 10.7–22.8 mIU/mL. FSH and testosterone rose modestly in parallel.

**Conclusion:**

While kisspeptin boluses have been employed to probe GnRH pulse generation, continuous infusion of kisspeptin is a complementary physiologic tool used to study dynamic changes in GnRH-induced LH secretion. Further studies are warranted to further elucidate the physiologic response of the kisspeptin receptor to non-pulsatile ligand administration.

**Clinical trial registration:**

NCT01438073 (August 24, 2011).

## Introduction

Kisspeptin plays a central role in GnRH secretion and is thus considered a key regulator of reproductive function. Within the arcuate nucleus, a specialized subset of kisspeptin neurons, known as KNDy (kisspeptin/neurokinin B/dynorphin) cells, functions as a “pulse generator,” coordinating rhythmic bursts of activity that drive the pulsatile release of GnRH [3, 4]. This finely tuned system is essential for normal sexual maturation, as evidenced by the consequences of genetic disruptions: loss-of-function mutations in the kisspeptin receptor (*KISS1R*) [5, 6] and kisspeptin gene (*KISS1*) [7, 8] are associated with hypogonadotropic hypogonadism, while gain-of-function mutations are associated with central precocious puberty [9, 10].

Because kisspeptin acts directly upstream of GnRH neurons, exogenous kisspeptin administration has emerged as a valuable tool for probing GnRH neuronal function. Much has been learned about the effects of acute kisspeptin administration in stimulating GnRH-induced LH secretion across a range of conditions, including healthy men [11, 12], healthy women [13, 14], individuals with congenital [15–17] or acquired hypogonadotropic hypogonadis [18, 19], and adolescents with delayed puberty [20, 21]. While the effects of kisspeptin administration via long infusion remain less clear, studies using kisspeptin analogs in human have shown that sustained exposure can suppress the reproductive axis [22, 23]. This phenomenon, akin to the downregulation observed with GnRH analogs at the pituitary level, opens avenues to potential therapeutic applications for hormonal modulation. However, the specific conditions under which a continuous kisspeptin infusion induces receptor desensitization in humans have yet to be fully determined.

This study aims to elucidate the effects of a continuous kisspeptin infusion on LH secretion in healthy men. To this end, we administered intravenous kisspeptin at 12.5 mcg/kg/h for 24 h to healthy adult men and performed frequent blood sampling before, during, and after the infusion to characterize its effects.

## Materials and methods

### Subjects and eligibility criteria

Three men participated in this study. They met the following inclusion criteria: 21–40 years old: self-reported history of normal timing of puberty and normal erectile and ejaculatory function; no use of prescription medications for at least 2 months before the study; body mass index 18.5–30 kg/m2; blood pressure below 140/90 mm Hg; normal physical examination including testicular volume 15 ml or greater by Prader orchidometer; normal white blood cell and platelet counts, normal hemoglobin; no elevation of creatinine or blood urea nitrogen; aspartate aminotransferase and alanine aminotransferase no more than twice the upper limit of the reference range; and normal TSH, prolactin, FSH, LH, and testosterone.

All protocols were approved by the Institutional Review Board of the Massachusetts General Hospital (MGH) and the Food and Drug Administration and all subjects gave written informed consent before participation in these studies. This study was registered with ClinicalTrials.gov no. NCT01438073.

### Materials and methods

Kisspeptin 112–121 was provided as kisspeptin-10 under contract to the Eunice Kennedy Shriver National Institute of Child Health and Human Development and was synthesized using good manufacturing practices by PolyPeptide Laboratories (San Diego, CA, USA). Resuspended aliquots underwent additional tests for purity, concentration, sterility, and pyrogenicity.

### Study design

Participants were admitted to the Translational Clinical Research Center at MGH for a 36-h study, with all visits standardized to a 7 a.m. to 7 p.m. schedule to minimize circadian rhythm variations.

#### Pre-infusion baseline (hours 0–6):

Subjects underwent blood sampling every 10 min to characterize baseline LH pulsatility in the absence of any peptide.

#### Kisspeptin infusion (hours 6–30):

Kisspeptin-10 was administered as a continuous intravenous infusion at 12.5 mcg/kg/h (*9.6 nmol/kg/h*) for 24 h. This dose was selected based on prior human studies demonstrating that a lower dose of 4 mcg/kg/h (*3 nmol/kg/h*) produced LH stimulation without attenuation, suggesting it was sub-threshold for desensitization [24]. A higher dose was therefore employed to increase the likelihood of observing kisspeptin receptor modulation over the 24-h period. Blood sampling continued every 10 min at the start (hours 6–12) and the end (hours 22–30) of the infusion, capturing the dynamics most informative for assessing attenuation. Between hours 12 and 22, sampling frequency was reduced to once per hour to comply with blood-drawing safety guidelines.

#### Post-infusion (hours 30–36):

The infusion was stopped at hour 30 and blood sampling at 10-min intervals resumed for a further 6 h to capture the recovery of endogenous pulsatile LH activity.

### Laboratory assays

During outpatient screening for eligibility, FSH, LH, and total testosterone (LC/MS) were measured at LabCorp using the Roche Elecsys system. For all research visits, samples were analyzed by the Massachusetts General Hospital (MGH) Clinical Laboratory Research Core using the automated Abbott ARCHITECT system. LH was measured at 10-min intervals, while FSH and testosterone were determined from 2-h study pools constituted from equal aliquots of the 10-min samples. All samples from a single study visit were analyzed together. The limits of detection for LH, FSH, and testosterone were 0.1 mIU/mL, 0.05 mIU/mL, and 10 ng/dl, respectively. Commercial QC controls confirmed intra-assay CV < 5% for LH and FSH. Study-specific assay precision was additionally monitored using a segmental QC pool representing baseline, infusion, and post-infusion periods; the intra-assay CV for LH was < 12%.

A validated modification of the method of Santen and Bardin was used to identify LH pulses [25]. To minimize false positive detections, every identified peak was required to exceed the local intra-assay CV by a factor of three. This pulse detection methodology has been previously validated on datasets with similar properties and sampling frequencies [12, 26].

### Statistical analysis

This study was a within-subject time-series physiologic experiment with *n* = 3 participants. Given the small sample size, no inferential statistics or *p*-values were calculated; instead, descriptive summaries are reported for each individual.

Hormone concentrations are reported as median (inter-quartile range, IQR: 25th–75th percentile) over the specified period for each participant. For FSH and testosterone, IQR is reported during baseline but not for other time periods, where values represent single 2-h pooled measurement. Four time periods are defined, as follows: (1) Pre (0–6 h): median concentration prior to infusion; (2) Max: highest 2-h rolling average LH (i.e., the highest average LH value across all possible consecutive 2-h windows during the infusion) or highest single pooled value (FSH, testosterone) during the infusion; (3) End (28–30 h): median concentration during the final 2 h of infusion; and (4) Post (34–36 h): median concentration after the infusion had ended, during the final 2 h of the study. For each subject, the following metrics were also derived: (a) time-to-max LH (hours from infusion start to the start of ‘max’); (b) max fold-change relative to pre-infusion baseline; and (c) percent decline from ‘max’ to ‘end’.

LH pulsatility parameters were assessed during pre-infusion, infusion, and post-infusion periods: pulse amplitude (height above nadir, mIU/mL), pulse frequency (number of pulses per 6 h), interpulse interval (pulse peak-to-peak interval) and pulse area under the curve (AUC). Parameters are presented as the mean of all pulses for each period. LH pulse AUC was calculated for each identified pulse using the trapezoidal method with 10-min sampling intervals. For each pulse, the area under the LH concentration–time curve was computed from the pre-pulse nadir to the post-pulse nadir, with a linearly interpolated baseline between these two nadirs subtracted to isolate the secretion attributable to that pulse.

## Results

The three male participants were aged 23 to 32 years and had a BMI ranging from normal weight to overweight (22.5 to 27.5 kg/m^2^). All participants had normal testicular volume, ranging from 20 to 25 mL, suggesting normal sexual maturation. Testosterone concentrations ranged from 578.7 to 640.8 ng/dL, within the normal range for men, in the setting of normal LH and FSH concentrations, indicating an intact hypothalamic-pituitary–gonadal axis. Baseline clinical and laboratory characteristics for each subject are summarized in Table 1.

During the ‘pre’ period, LH concentrations across participants ranged from 2.8–3.8 mIU/mL (Table 2). Pulsatile secretion was present in all subjects at a frequency of 2 to 3 pulses per 6 h, consistent with normative data in healthy adult men [27]. Baseline mean pulse amplitude ranged from 1.1–2.8 mIU/mL and mean pulse area from 23–159 mIU/mL·min across participants (Table 3).

**Fig. 1:**
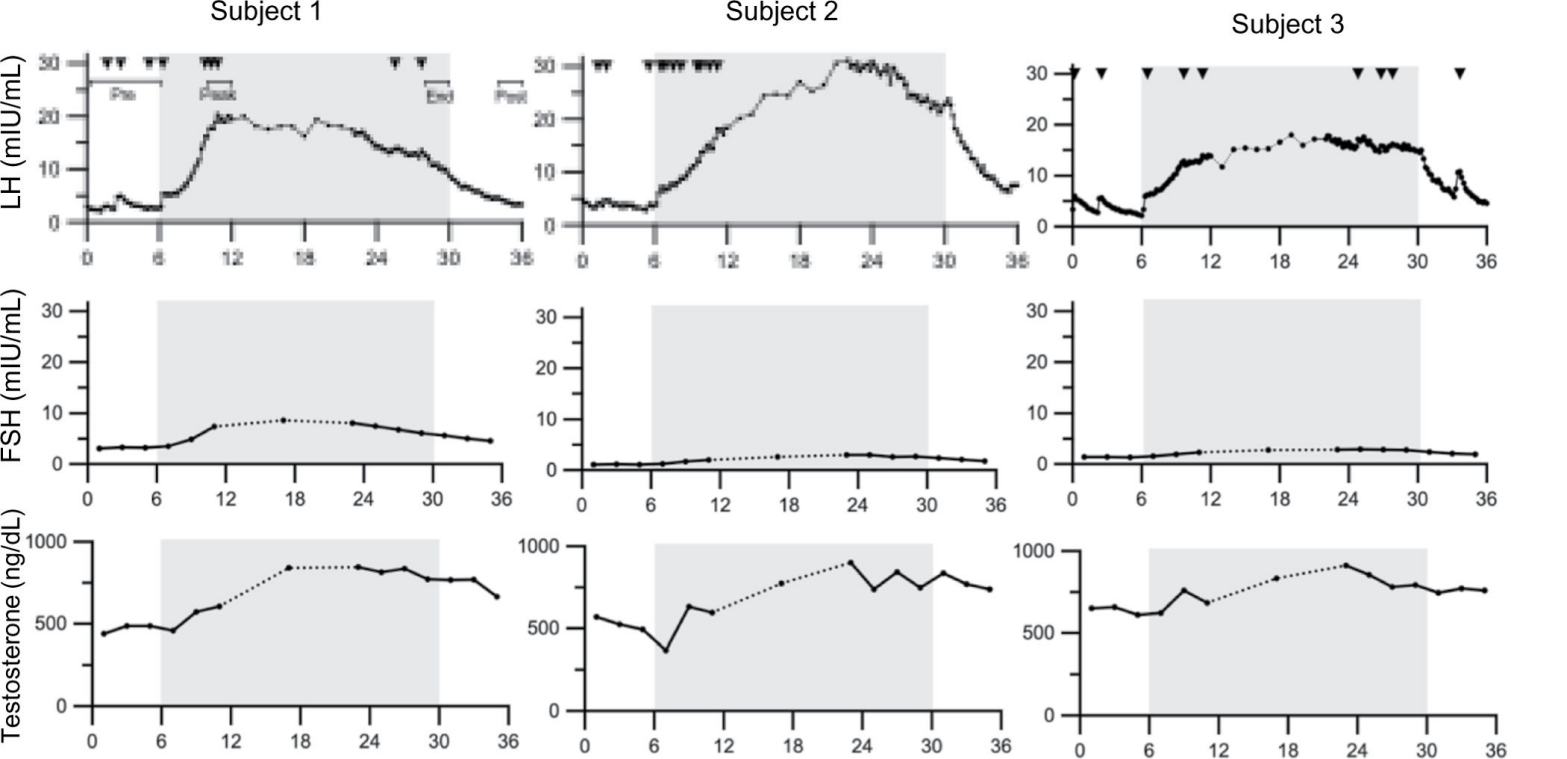
LH, FSH and testosterone concentrations before, during and after kisspeptin administration. *Shaded box* indicates the 24-h kisspeptin infusion period (*12.5 mcg/kg/h*); *arrowhead* indicates peak of individual LH pulses. *Dotted lines* represent the reduced blood sampling interval

Following initiation of the kisspeptin infusion, LH concentrations rose in all three participants (Fig. 1; Table 2). ‘Max’ LH concentrations of 17.5–30.6 mIU/mL were reached within 11.6–20.3 h of infusion onset, representing 5.1- to 8.0-fold increases above individual baselines. Subsequently, LH concentrations had a variable decline across all subjects (47%, 25%, and 13% in Subjects 1, 2, and 3, respectively). Moreover, ‘end’ LH concentrations remained above ‘pre’ in all subjects, indicating that partial rather than complete attenuation occurred over 24 h.

Following cessation of the infusion, LH concentrations declined further over the subsequent 6 h. Subject 3 had one discrete pulse detectable during the post-infusion period, whereas Subjects 1 and 2 showed no detectable pulses, suggesting that restoration of pulsatile LH secretion may not be immediate in all individuals. ‘Post’ LH concentrations of 3.8–7.4 mIU/mL were still above ‘pre’ LH concentrations of 2.8–3.8 mIU/mL, with variable elevation across subjects (+ 36%, + 94%, and + 59% in Subjects 1, 2, and 3, respectively).

Table 3 describes LH pulse parameters during pre-infusion, infusion, and post-infusion for each participant. Notably, LH pulse parameters during infusion should be interpreted cautiously, as the continuously shifting LH baseline impacts pulse detection and likely underestimates the number of identified pulses. Subject 1 showed a slightly lower mean pulse frequency during infusion compared to pre-infusion (3 → 2.6 pulses/6 h), with pulses of larger mean amplitude (1.2 → 2.3 mIU/mL) but lower mean AUC (70 → 37 mIU/mL·min). Subject 2 showed a higher pulse frequency (3 → 3.4 pulses/6 h), with a significantly reduced interpulse interval (125 → 40 min), and pulses of greater mean amplitude and AUC (1.1 → 2.1 mIU/mL and 23 → 56 mIU/mL·min, respectively). Subject 3 showed pulses of lower amplitude and significantly reduced AUC during infusion (2.8 → 1.9 mIU/mL and 159 → 31 mIU/mL·min, respectively), with a slightly higher pulse frequency (2 → 2.6 pulses/6 h). Overall, these findings demonstrate inter-individual variability in LH pulsatility parameters in response to kisspeptin infusion.

FSH and testosterone concentrations rose in parallel with LH. FSH rose from ‘pre’ concentrations of 1.1–3.2 mIU/mL to ‘max’ concentrations of 2.9–8.7 mIU/mL, representing 2.1- to 2.7-fold increase above baseline, much lower than seen with LH. At ‘end’ infusion, FSH remained elevated at 2.4–5.7 mIU/mL, mirroring the partial attenuation pattern seen with LH. Testosterone peaked at 847–913 ng/dL across subjects, lagging behind the LH ‘max’ in keeping with the time required for Leydig cell steroidogenesis, and remained elevated above baseline through the end of the infusion (747–836 ng/dL; Table 2).

## Discussion

We have demonstrated that intravenous administration of kisspeptin-10 at 12.5 mcg/kg/h (*9.6 nmol/kg/h*) over 24 h in healthy men induced an initial rise in LH concentrations, followed by a plateau and a subsequent decline towards the end of the infusion across all participants. Notably, while continuous kisspeptin infusion did not suppress LH, the observed changes in gonadotropin secretion suggest that continuous intravenous kisspeptin administration has the capability to modulate GnRH, and by extension LH release.

Fifty years ago, the Knobil laboratory demonstrated that in hypothalamically-lesioned rhesus monkeys, intermittent administration of GnRH reestablished pituitary gonadotropin release, but a constant infusion failed to restore sustained gonadotropin secretion [28]. This seminal work pointed to the importance of the pattern of GnRH delivery, as opposed to simply the dose, in regulating reproductive function. Moreover, it suggested that continuous GnRH administration could result in downregulation of the signaling pathways responsible for gonadotropin release. Over the years, this phenomenon, termed “desensitization”, has been described as a waning of the receptor response in the face of continuous agonist exposure [29].

Subsequent human and animal GnRH infusion studies have provided insights into gonadotropin desensitization. During 72-h GnRH infusions in healthy men [30] and follicular phase women [31], LH concentrations peaked at approximately 24 h, then declined but remained 3–4 times above baseline by the end of the infusion. Following GnRH’s discovery, more potent analogs were developed, which, like native GnRH, initially augmented LH but eventually suppressed gonadotroph and gonadal function. In one study of healthy men using chronic buserelin infusion via osmotic minipumps, LH concentrations sharply declined after the initial peak by the end of first week with further suppression in LH and testosterone over subsequent weeks [32]. Notably, subjects receiving the higher buserelin dose showed signs of desensitization as early as at 3 days, with an abolished gonadotropin response to a GnRH bolus during the infusion. Although kisspeptin may not behave identically to GnRH, these studies help contextualize the possible effects of continuous exposure of the kisspeptin receptor to the kisspeptin ligand or an analog.

Like GnRH, kisspeptin is a hypothalamic peptide derived from a larger precursor molecule [33] and is secreted in pulsatile fashion [34]. Continuous infusion of kisspeptin, as demonstrated in several animal species [1, 35–38], initially induces GnRH-mediated LH release, followed by a progressive decline in LH concentrations. However, notable differences in the observed patterns across species emphasize the complexity of the mechanisms. The time required for LH desensitization is variable between rats [36, 38], ewes [37], and monkeys [1, 35], ranging from 24 h to 2–3 days. Variations in kisspeptin response can be partly attributed to differences in experimental design, including kisspeptin peptide formulation, dosing, and duration of exposure. Underlying biological factors may also contribute to variability in kisspeptin response, with notable species differences in kisspeptin cell populations within the hypothalamus [39]. Finally, the response to kisspeptin can change depending on developmental stage and, by extension, sex steroid environment [40, 41].

While both GnRH and kisspeptin systems exhibit attenuation with sustained exposure, their desensitization profiles may differ in mechanism. GnRH receptor has a unique physical structure that lacks a cytoplasmic tail, which is typically required for rapid β-arrestin-mediated desensitization. Instead, GnRH receptor desensitization mainly reflects post-receptor adaptations—including diminished calcium signaling and transcriptional responses—during sustained exposure to its ligand [42, 43]. In contrast, the kisspeptin receptor does have a cytoplasmic tail, and its desensitization is mediated by classical G protein-coupled receptor mechanisms [44, 45], namely, receptor phosphorylation, β-arrestin recruitment, internalization, and receptor downregulation [46, 47]. Internalized receptors may be recycled or degraded, and sustained stimulation increasingly favors degradation, leading to long-term desensitization.

The concept of reduction in LH concentrations with continuous kisspeptin infusion reflects an effect at the level of the hypothalamus rather than the pituitary. Evidence for this comes from two studies in rhesus monkeys that employed a comparable experimental design, specifically, a continuous kisspeptin infusion over 98 h followed by sequential bolus challenges on Day 4 to probe the hypothalamic-pituitary axis. Seminara et al. first demonstrated this in GnRH-primed agonadal juvenile male rhesus monkeys: a kisspeptin bolus administered during a continuous infusion failed to elicit an LH rise, while boluses of NMDA and exogenous GnRH both produced robust LH responses, localizing the desensitization to the hypothalamus with preserved pituitary responsiveness [1]. Ramaswamy et al. subsequently extended this paradigm to adult eugonadal male rhesus monkeys: at lower doses, kisspeptin responses were abolished with preserved GnRH-stimulated LH release, while at higher doses, pituitary responsiveness to GnRH was also attenuated [2]. This pattern suggests that desensitization reflects reduced stimulatory kisspeptin input to GnRH neurons; however, it is still not fully understood how this diminished drive affects overall GnRH secretory dynamics. Our findings in adult eugonadal men may reflect the early desensitization trajectory similar to that observed in adult eugonadal male rhesus monkeys.

Human investigations into the downregulation of the reproductive axis in response to continuous kisspeptin administration have been limited and have yielded variable results. Several years ago, an important study demonstrated that twice-daily subcutaneous injections of kisspeptin-54 for 2 weeks in women with hypothalamic amenorrhea potently stimulated gonadotropin release at the start of study [48]. However, with continued administration, the effect diminished, providing one of the first pieces of evidence in humans that repeated stimulation of the kisspeptin receptor might lead to reduced LH release. Other studies using intravenous kisspeptin administration for shorter durations have revealed different biological responses. For example, an 8-h continuous infusion of kisspeptin-54 in women with hypothalamic amenorrhea increased LH pulses [49]. Studies examining kisspeptin-10 infusion at 4 mcg/kg/h (*3 nmol/kg/h*) in both healthy men [24] and men with type 2 diabetes mellitus [50] over 22.5 h demonstrated an initial rise in LH with increased LH pulsatility, but no decrease in LH concentrations.

In contrast, clinical studies utilizing oligopeptide analog of kisspeptin, TAK-448, administered subcutaneously (*0.01–1 mg/day which is approximately equivalent to 0.05–0.5 pmol/kg/h*) resulted in profound downregulation of the reproductive axis. When given to healthy male volunteers for 2 weeks, LH and testosterone concentrations fell to below-baseline values by 60 h and further declined to castration concentrations by day 8 [22]. This evidence for desensitization of the hypothalamic-pituitary–gonadal axis was achieved at all doses tested. Although direct comparisons have not been performed in human subjects to our knowledge, studies in rats have shown that TAK-448 decreases plasma LH and FSH concentrations more rapidly and effectively compared to the GnRH agonist leuprolide [23].

In summary, while our study reinforces previous observations that kisspeptin-10 infusion initially stimulates the hypothalamic-pituitary–gonadal axis, it remains unclear whether prolonged exposure duration will attenuate kisspeptin receptor signaling in the eugonadal male. Modulation of kisspeptin receptor signaling in humans is likely to be both time- and dose-dependent. As previous studies have varied in the isoform of kisspeptin used and their pharmacokinetics [51, 52], it is important to remember that kis-speptin-10 and −54 may differ with respect to thresholds for receptor saturation and desensitization. Our study was designed as a proof-of-concept experiment to define the physiological response to sustained kisspeptin exposure, which can inform future studies.

## Figures and Tables

**Table 1:** Baseline characteristics

	Subject 1	Subject 2	Subject 3	Reference range

Age, y	32	23	27	-
BMI, kg/m^2^	26.7	27.5	22.5	-
Testicular volume, mL	20	25	25	15–25
Creatinine, mg/dL	0.92	1.0	0.83	0.76–1.27
ALT, U/L	15	34	19	0–55
TSH, μIU/mL	2.7	0.9	3.6	0.45–4.5
Prolactin, ng/ml	9.1	8.1	9.1	4.0–15.2
LH, mIU/mL	3.7	5.0	3.1	1.7–8.6
FSH, mIU/mL	3.7	1.1	1.4	1.5–12.4
Testosterone, ng/dL	641	602	579	348–1197

*BMI* body mass index; *ALT* alanine transaminase; *TSH* thyroid-stimulating hormone; *LH* luteinizing hormone; *FSH* follicle-stimulating hormone

**Table 2:** Within-subject hormone concentration responses during 24-h continuous kisspeptin-10 infusion

	Subject 1	Subject 2	Subject 3

LH (mIU/mL)			
Pre (0–6 h)	2.8 (2.6–3.1)	3.8 (3.5–4.1)	3.4 (2.9–4.4)
Max (2-h window)	20.0 (19.8–20.1)	30.6 (30.2–30.9)	17.5 (17.2–17.8)
End (28–30 h)	10.7 (10.0–10.9)	22.8 (22.4–23.4)	15.3 (15.1–15.9)
Post (34–36 h)	3.8 (3.4–4.1)	7.4 (6.8–7.6)	5.7 (4.9–6.7)
Time to Max (h)	11.6	20.2	20.3
Max Fold-Change	7.1x	8.0x	5.1x
% Decline from Max to End	47%	25%	13%
% Change from Pre to Post	+ 36%	+ 94%	+ 59%
FSH (mIU/mL)			
Pre (0–6 h)	3.2 (3.2–3.3)	1.1 (1.1–1.2)	1.42 (1.3–1.4)
Max (2-h window)	8.7	3.0	2.9
End (28–30 h)	5.7	2.4	2.4
Post (34–36 h)	4.58	1.81	1.94
Testosterone (ng/dL)			
Pre (0–6 h)	486 (462–487)	524 (509–548)	651 (631–655)
Max (2-h window)	847	902	913
End (28–30 h)	768	836	747
Post (34–36 h)	666	740	760

Values are given as median (IQR). IQR represents the 25th–75th percentile range of hormone concentrations per participant

FSH and testosterone max, end, and post infusion values are single pooled measurement; IQR is not applicable

**Table 3:** LH pulsatility parameters during pre-, infusion, and post- kisspeptin-10 infusion periods

	Mean pulse amplitude (mIU/mL)			Mean pulse frequency (no. of pulses in 6 h)			Mean interpulse Interval (min)			Mean pulse AUC (mIU/mL · min)		
			
	Pre	Infusion	Post	Pre	Infusion	Post	Pre	Infusion	Post	Pre	Infusion	Post

Subject 1	1.2	2.3	-	3	2.6	0	105	100	-	70	37	-
Subject 2	1.1	2.1	-	3	3.4	0	125	40	-	23	56	-
Subject 3	2.8	1.9	5.0	2	2.6	1	140	118	ND	159	31	274

Dash indicates no pulse detected in the specified period; ND indicates interpulse interval could not be calculated (fewer than 2 pulses detected)

## Data Availability

The datasets generated during and/or analyzed during the current study are available from the corresponding author on request.
